# Comparing the effect of plasma therapy with estradiol valerate in motor and cognitive behavioral disorders in ovariectomized old rats: Behavioral, biochemical, and protein expression

**DOI:** 10.22038/ijbms.2024.81345.17608

**Published:** 2025

**Authors:** Maryam Khajvand-Abedini, Mohammad Mohammadi, Parisa Habibi, Zahra Shahabi, Siamak Shahidi, Nasser Ahmadiasl, Mohammad Reza Alipour, Mahdi Ramezani, Alireza Komaki

**Affiliations:** 1 Department of Clinical Biochemistry, Faculty of Medicine, Hamadan University of Medical Sciences, Hamadan, Iran; 2 Department of Medical Laboratory Science, Faculty of Medicine, Kermanshah Branch, Islamic Azad University, Kermanshah, Iran; 3 Department of Physiology, School of Medicine, Tehran University of Medical Sciences, Tehran, Iran; 4 Neurophysiology Research Center, Hamadan University of Medical Sciences, Hamadan, Iran; 5 Stem Cell Research Center, Tabriz University of Medical Sciences, Tabriz, Iran; 6 Department of Anatomy, School of Medicine, Hamadan University of Medical Sciences, Hamadan, Iran

**Keywords:** Cognition, Estradiol valerate, Motor, Ovariectomy, Protein expression, Young plasma

## Abstract

**Objective(s):**

This study investigated the effects of young plasma therapy (YPT) compared to estrogen therapy (E2T) on motor and cognitive impairments in aged ovariectomized (OVX) rats.

**Materials and Methods:**

Sixty female Wistar rats were divided as follows: 1). 2-3 months control young group. Five 22-24 months old groups: 1) Control, 2) Sham, 3) OVX, 4) OVX.E2, and 5) OVX.YP. Young plasma (1 ml plasma, through the tail vein, 3 days weekly for 4 weeks) and E2 (30 mg/kg, SC, 5 days weekly for 4 weeks) were administrated to OVX rats. The open field, elevated plus maze, and Barne’s maze were used to assess the behaviors. Then, miR-134 and miR-124 (RT- RCR), SIRT1, CREB, and BDNF (western blot), and anti-oxidants/oxidants markers (Photometry) levels were assessed in the rat’s hippocampal tissues.

**Results:**

OVX caused up-regulated hippocampal miR-134 and miR-124 expression levels (*P*<0.001) while down-regulated SIRT1, CREB, and BDNF protein expressions (*P*<0.001). Also, ovariectomy Increased TOS, OSI, and MDA (*P*<0.001) while decreasing TAC (*P*<0.001) compared to sham. Treatment with both E2T and YPT significantly improved all oxidative stress indexes (*P*<0.0.001) and increased p-CREB, BDNF, and SIRT1 protein levels (*P*<0.05, *P*<0.01) while decreasing the expression of miR-134 and miR-124 (*P*<0.001).

**Conclusion:**

YPT is a non-pharmacological therapeutic as much as or more than E-2T, which can exhibit anti-oxidative and anti-inflammatory potential in the hippocampal tissue and improve cognitive deficits in aged OVX rats without unknown side effects.

## Introduction

Aging changes in the female reproductive system are associated with major health problems such as obesity, depression, anxiety, neurological diseases, and cognitive disorders (1). 17β-estradiol or estrogen-2 (E2), the primary ovarian hormone, exerts neuroprotective effects (2). The lack of E2 with reproductive senescence, followed by decreased hippocampal function and plasticity and increased anxiety, occurs in aged patients (3). Also, the elderly brain is damaged by oxidative stress because of the low activity of anti-oxidant enzymes and the high utilization of inspired oxygen (4). Based on animal studies, ovariectomized (OVX) animals are at remarkably complex risk for age-related dementia and cognitive disorders (5), and estrogen has beneficial impacts on mental abilities and anxiety-like behaviors in these animals (6). It was reported that E2 therapy (E2T) ameliorated cognitive and memory decline of elderly rats by upregulation of CA1 hippocampal brain-derived neurotrophic factor (BDNF) and phosphorylated form of cAMP-responsive element-binding protein (p-CREB) (7). Likewise, E2 increased the sirtuin1 (SIRT1) expression and activation of AMP kinase in cerebral neurons *in vitro* (8). During aging, SIRT1 is in control for the care of neural systems and behavior, including the variation of synaptic plasticity and memory progressions, and deficit of SIRT expression in the hippocampal neurons will decline cognitive function, as well as recent spatial learning and memory (9). 

Among all neurotrophins, brain-derived neurotrophic factor (BDNF) stands out owing to its high level of expression in the brain and its possession of synapses (10). The hippocampus, one of the brain areas complicated in memory consolidation, is mainly sensitive to oxidative stress that leads to neuronal decease (11). Diminished expression of some brain factors in the hippocampus, such as brain-derived neurotrophic factor (BDNF), cAMP-responsive element-binding protein (CREB), and sirtuin-1 protein (SIRT1) is intricate in learning and memory disorders (12).

MicroRNAs, small non-coding RNAs, contribute to numerous features of hippocampus physiology, such as growth, neurogenesis, and synapse formation (13). MiR-134, miR-146-5p, miR-138, miR-8-5p, miR-124, miR-137, and miR-184 are the greatest studied miRNAs in the hippocampus (14). Eyileten, in a study in 2021, presented that miR-134a can target BDNF genes and disturb that expression (15). The NAD-dependent deacetylases SIRT1 are vivacious for learning, and this function requires collaboration with brain-specific miR-134. SIRT1 suppresses transcriptional regulation of miR-134, and miR-134 expression is up-regulated in SIRT1-deficient mice. Overexpression of miR-134 adjusts negative memory foundation in rodent hippocampus concluded translational repression of CREB mRNA (16, 17).

Recent studies demonstrated that SIRT1 improved hippocampal-related memory and cognitive functions (8) by down-regulation of miR-134 (17). Unchecked miR-134 appearance following SIRT1 deficit results in the downregulated expression of CREB and BDNF, thereby weakening synaptic plasticity (12). Overexpression of miR-124 inhibits the expression of SIRT1 and promotes the generation of ROS (18). Parallel to miR-124, the overexpression of miR-134 negatively regulates fear memory formation and long-term potentiation induction in the rodent hippocampus concluded translational repression of CREB mRNA; so, it affects memory and learning by mediating the CREB-BDNF-dependent signaling pathway (16). E-2T, as hormone replacement therapy, is the best option to attenuate indications of menopause in the aging period (19). However, supplementary research has reported several disadvantageous effects of E2T utilization for a long time, such as an increased risk of stroke, endometrial, or breast cancer (20).

Therefore, an alternative treatment could be more effective than E2T with destructive side effects. In the last decade, studies have used parabiosis as an alternative therapeutic method for age-related diseases (21, 22). It was conducted by sharing the bloodstream between animals (senile to senile or young to young (isochronic), young to senile (heterochronic)) (23). Conese, in 2017, showed that the parabiosis method and factors in young blood by stimulating vascular regeneration lead to increased neurogenesis and olfactory diagnosis in older mice (24). Another study also showed the re-myelination of old mice through heterochronic parabiosis due to the possible passage of young plasma over the blood-brain barrier (BBB) to the central nervous system (25). The administration of human umbilical cord plasma was reported to improve anxiety and cognitive impairments in old mice (26). The circulatory factors in young blood can converse age-related cognitive debits and return synaptic plasticity by reversing age-related cognitive debits in mice (27). One study in 2024 discovered that small extracellular vesicles from young plasma reverse deteriorating deviations and age-related dysfunction by motivating PGC-1α expression and growing mitochondrial energy metabolism (28). However, the effects of YPT on menopause-related cognitive and anxiety dysfunctions during aging have not yet been indicated. So, in the present study, we assumed that YPT on old OVX rats would reduce anxiety and cognitive disorders and have anti-oxidant impacts. Accordingly, we have assessed the following actions: 1) Can YPT effectively treat cognitive deficiencies? 2) Are young plasma like E2 impressive on behavioral examinations in the Elevated Plus maze, Open field, and Barnes’s maze tests? 3) Does young plasma like E2 reduce oxidative damage in the CA1 region of the hippocampus? 4) Will young plasma like E2 ameliorate anxiety and cognitive impairments by amending the level of cell signaling factors?

## Materials and Methods

### Study design

**Figure 1:**
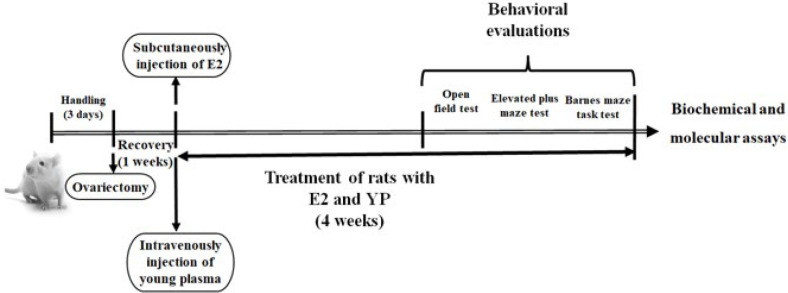
Experimental timeline using a rat model

Fifty old female Wistar rats (22-24 months, 300–350 g, into five groups) and ten young (2-3 months, 180–220 g, into one group) were prepared. Additionally, 30 young rats (2-3 months, 180–220 g, 15 males; 15 females) were gained from the Hamadan University of Medical Sciences for plasma collection. The animals are held in standard situations under a 12-hr light / dark cycle with *ad libitum* access to food and tap water. The application of animal surgery, treatment procedures, and care methods of laboratory animals in our study was approved by the institutional ethics committee at Hamadan University of Medical Sciences (IR.UMSHA.REC.1397.638). The rats were randomly partitioned into six groups as follows:

1. YC: Young control group: without bilateral ovariectomy surgery and received vehicle. 

2. OC: Old control group: without bilateral ovariectomy surgery and received vehicle. 

3. Sham: Aged rats who underwent surgery but without bilateral ovariectomy and received vehicle. 

4. OVX: Aged rats underwent two-sided ovariectomy without treatment (29) and received vehicle. 

5. OVX.E2: Aged rats underwent situations like group OVX with 17β-estradiol (ER-2) administration [30 mg/kg, subcutaneously, 5 days weekly for 4 weeks; (Bayer HealthCare Pharmaceuticals, Berlin, Germany) (30)]. 

6. OVX. YP: Old rats underwent situations like group OVX with young plasma administration (1 ml/daily, intravenously injection through the tail vein, 5 days weekly for 4 weeks) (31). The investigational timeline is shown in Figure 1.

### Ovariectomy

After the rats were anesthetized with a mixture of ketamine-xylazine (90/10 mg/kg), a slight incision was made on both sides of the dorsal region of the rat body, and the ovaries were separated after closure. In the sham group, the only surgical incision was made without ovarian removal (32).

### Young plasma collection

The blood departed through the inferior vena cava as of anesthetized the young (2-3 months old) rats. Plasma was achieved by centrifugation (1000×g10 min) from sodium citrate and blood mixture. Plasma from female and male rats was blended at 1:1 (vol/vol). Thereafter, plasma aliquots were kept at -80 °C (27).

### Behavioral evaluations

***Open field (OF) test***

Twenty-four hours after the end of treatments, the locomotor activity of rats was assessed in the open field test (50 cm × 50 cm × 40 cm, MazeRouter Co. Tabriz, Iran) for 5 min. The rat’s velocity and total travel distance were tracked via a video camera and analyzed using the MazeRouter software (33).

### Elevated plus maze (EPM) test

Another behavioral test was applied to diagnose anxiety-like behavior. EPM device (MazeRouter Co. Tabriz, Iran) is composed of two closed arms (10×50×30) and two open arms (10×50×0.5 cm) that intercross at the centric, creating a plus shape. Furthermore, the EPM apparatus was raised 50 cm above the floor. Briefly, during the 5-minute test, the animal was located in the central area and opposite the open arms to probe the apparatus. After recording the rat’s movements with the camera, the time used up in the two open arms, and the number of entries into them was tracked and analyzed (34). 

### Barnes maze task (BMT) test

BMT is a standard experimental method to evaluate spatial learning and memory (35, 36). The BMT period consisted of four training days and one test day with the probe, working 1 and working 2 trials (each trial spent 3 min). BMT apparatus was placed in a closed surrounding area with four different arrow shapes on the walls. BMT apparatus comprised a circular platform with eighteen holes in its edges. Moreover, the circular platform was divided into four quadrants (Q1, Q2, Q3, and Q4), and a black box was placed under one hole of the target quadrant (Q2) for the escape of rats on training days. After training, on test day, the box was removed, and in working trials (working 1 and 2), the box was located in Q4 (180 degrees far from Q2) in working 1; afterward, it was removed in working 2. During BMT training with three trails, a noise sound was played and stopped as soon as they found the box animals. In contrast, the noise was played continuously and without stopping in the probe and working two trials (without a box). Finally, the latency time each rat spent entering the box (recorded by the video tracking camera) and the counted errors (dipping the nose into holes that did not associate with the black box) were assessed.

### Biochemical evaluations

Following behavioral tests, rats were anesthetized with a blend of ketamine/xylazine (90:10 mg/kg). Four rats per group were applied for the transcardial perfusion technique and, finally, histopathological evaluations. Six rats per group were applied in molecular and biochemical analysis. Furthermore, after departing and cleaning the rat’s hippocampus with ice-cold saline, it is speedily frozen in fluid nitrogen and stored at –80 °C. 

### Oxidative stress evaluations

After homogenizing the hippocampus in lysis buffer and a protease inhibitor cocktail (Kiazist Life Sciences, Iran), the acquired supernatant was saved at –70 ◦C. The index of lipid peroxidation, known as total malondialdehyde (MDA), was determined using an MDA kit (Kiazist, Iran). Total anti-oxidant capacity (TAC) was assessed by ferric reducing anti-oxidant power reagent and related method, while total oxidant status (TOS) was evaluated by oxidation of ferrous (Fe 2+) to ferric (Fe3+). Additionally, the oxidative stress index (OSI) was calculated by the division of TOS to TAC. The hippocampus’s total protein content was assessed through the Bradford method, and standard bovine serum albumin (BSA) was applied as standard (37).

### Western blot evaluations

The Radio-immunoprecipitation assay (RIPA) buffer with the protease inhibitor was utilized to prepare the tissue homogenates. The bicinchoninic acid (BCA) method was applied to evaluate the total protein content of tissues. Furthermore, sulfate-polyacrylamide gel electrophoresis (SDS-PAGE) was utilized to fractionate homogenates, then conveyed to membranes with nitrocellulose genus. After obstructive, the primary antibodies, SIRT1, BDNF (108319, Abcam), phosphorylated and activated form of CREB (p-CREB), CREB, and GAPDH as housekeeping protein (sc-74504, SC- 7978, sc-377154, and sc-32233 respectively, Santa Cruz Biotechnology) were utilized. In addition, density values were calculated by using Image J software (38). 

### MicroRNA-134 and microRNA-124 expression evaluations

Hippocampal microRNA-134 (miR-134) and microRNA-124 (miR-124) expression stages were measured by quantitative RT-PCR. Briefly, tissue total RNA was extracted using Roche TriPure reagent (# 116671657001; Roche, Mannheim, Germany). After assessing quantified by a NanoDropTM instrument (Thermo Fisher Scientific, USA) and assessing qualified by agarose gel electrophoresis (1%), cDNA was synthesized by Prime Script RT reagent kit (TaKaRa Biotechnology, Japan). RT-PCR was done via SYBR Green master mix (Amplicon, Denmark) on the LightCycler_96 apparatus (Roche Life Science Deutschland GmbH, Sandhofer, Germany). Primers were depicted in Table 1. At last, it should be mentioned that U6, as a housekeeping gene, and the 2^− ΔΔCt^ method were secondhand for relative gene appearance (fold change) (39). 

### Statistical evaluations

All statistical evaluations were done using the SPSS software type 16 (SPSS Inc., Chicago, USA) and GraphPad Prism type 8.0. Data values were expressed as mean ± SEM, and *P-values*<0.05 were considered statistically significant. The one-way ANOVA followed by Tukey’s *post-hoc* test was used to compare alterations between investigational groups.

## Results

### Results of behavioral evaluations

***Locomotion***

**Figure 2:**
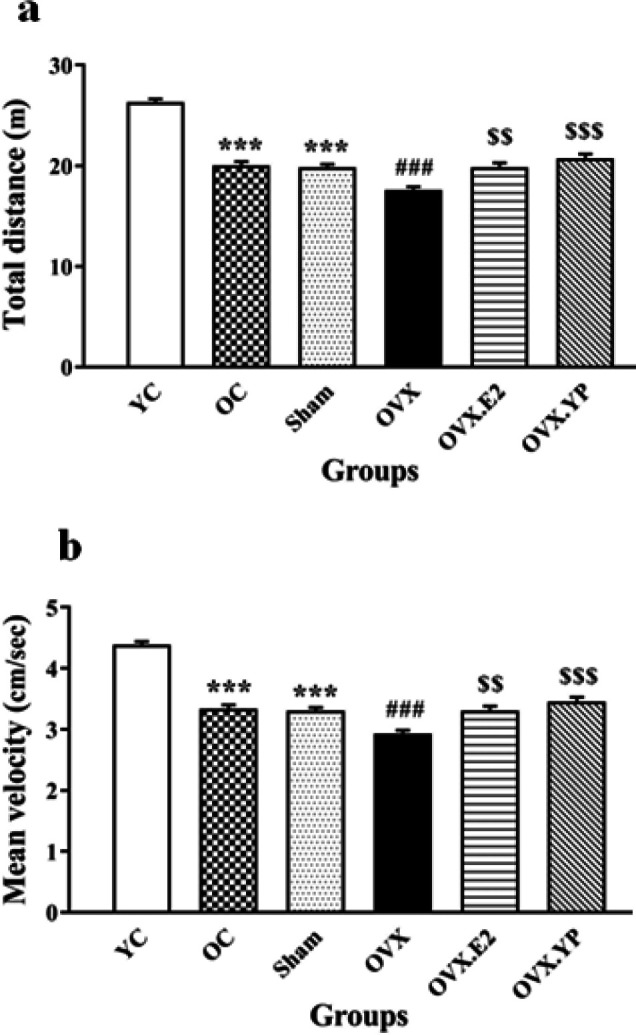
Outcome of young plasma taking place in the open field (OF) (a-b) in ovariectomized old rats. a) Total travel distance and b) Mean velocity in the OF test

The results of the total distance traveled tested in the open field are shown in Figure 2a [F (6, 30) = 66.97, (*P*<0.0001)]. 

This locomotion parameter was decreased significantly in OC, Sham, and OVX groups compared to YC (*P*<0.001). Additionally, total distance significantly declined OVX compared to OC and Sham groups (*P*<0.001, *P*<0.01) while increasing in OVX.E2 and OVX. YP groups related to OVX (*P*<0.01, *P*<0.001). 

Moving velocity is shown in Figure 2b [F (6, 35) = 100/3, (*P*<0.0001)]. 

The locomotion speed decreased significantly in OC, Sham, and OVX groups compared to YC (*P*<0.01, *P*<0.001). It significantly declined OVX compared to OC and Sham groups (*P*<0.001), while it increased in OVX.E2 and OVX. YP groups related to OVX (*P*<0.001). The speed of locomotion in OVX rats treated with YP (OVX.YP) was higher than OVX.E2 (*P*<0.001).

***Anxiety-like behavior***

**Figure 3:**
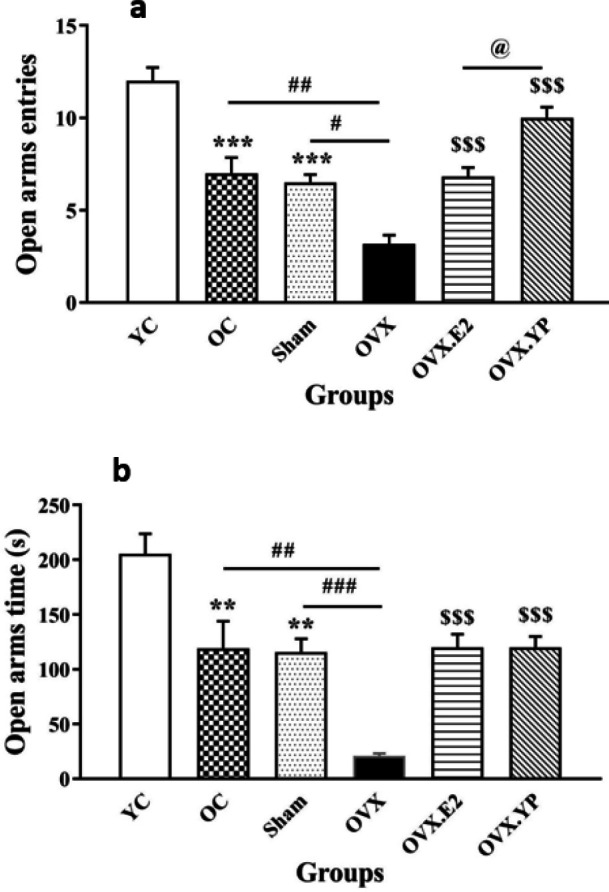
Effect of young plasma on the elevated plus maze (EPM) (a-b) tests in ovariectomized old rats. c) Number of entries into open arms. d) Time spent in the open arms (sec)

The open arms entries (OPE) in the elevated plus maze (EPM) are shown in Figure 3a [F (5, 30) = 25.15, *P*<0.0001). 

OPE significantly decreased in OC and Sham groups associated with YC (*P*<0.001). The OVX group had a significant reduction in the OPE compared to the OC and sham groups (*P*<0.01). Results showed that OVX.E2 and OVX. YP groups had significantly increased entries into the open arms compared to OVX (*P*<0.01, *P*<0.001). Furthermore, the OVX. YP group illustrated a significant rise in the number of entries into the open arms related to the OVX.E2 group (*P*<0.05). 

The time used up in open arms (OPT) of EPM is shown in Figure 3b [F (5, 30) = 15.20, *P*<0.0001). 

Compared to YC, OPT was significantly decreased in OC, Sham, and OVX groups (*P*<0.01, *P*<0.001). The OVX group had a significant reduction in the OPT (*P*<0.001) compared to OC. Results showed that OVX.E2 and OVX. YP groups had significantly increased OPT compared to OVX (*P*<0.001). The OPT in OVX. YP group had no significant difference with OVX.E2.

### Barne’s maze task (BMT)

**Figure 4:**
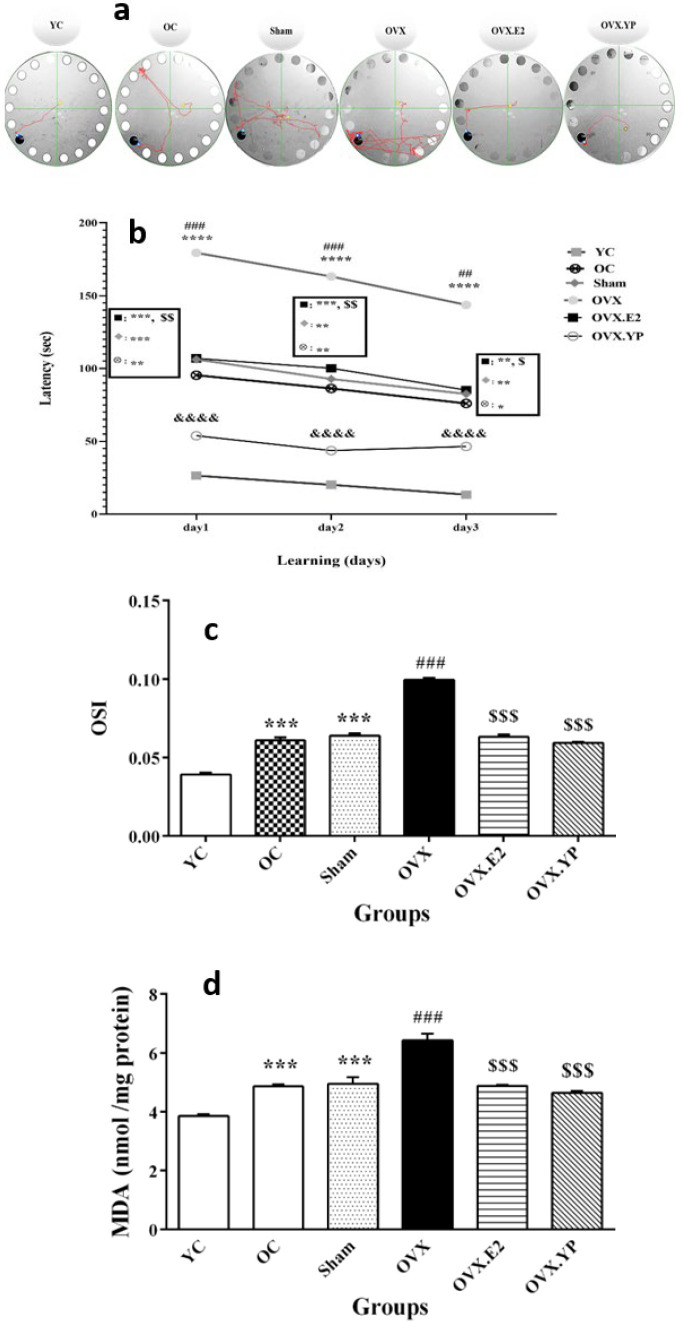
Result of young plasma on the Barnes maze task (BMT) in ovariectomized old rats

Analysis of tracking, latency [F (5, 30) = 13.84, *P*<0.0001), numeral of errors [F (5, 30) = 12.21, *P*<0.0001)], and time used up in the goal quadrant in BMT are shown in Figures 4a-e. 

The tracking patterns are shown in Figure 4a. Analysis of data indicated that rats in OC and sham groups had notably increased latency on the first to third days of the training period (*P*<0.05, *P*<0.01, Figure 4b) and the number of errors (*P*<0.05, Figure 4c) compared to YC group. OVX significantly increased escape latency and error numbers on all three days of the training period (*P*<0.001, Figures 4a-c) compared with OC *post-hoc* comparisons revealed that both treated groups (OVX.E2 and group OVX.YP) had remarkably decreased error numbers (*P*<0.001, Figure 4c) and escape latencies (*P*<0.001, Figure 4b) against the OVX group at all of the training days. They were following the comparison of the OVX.E2 group with the YC group, the OVX. YP group had a substantial decrease in the escape latency and the error numbers on the first, second, and third days of training (*P*<0.001, *P*<0.01, *P*<0.05 Figures 4b-c).

Also, analysis of the time used up in the Q2 and the error numbers in the probe trial to find the target box showed that rats in OC and sham groups had a notable decrease (*P*<0.05, *P*<0.01 Figures 4d and e). OVX had lower time spent (*P*<0.01, Figure 4d) while the increased number of errors (*P*<0.01, Figure 4e) in the target quadrant than the OC and sham groups. After treatment with estrogen and young plasma, there was a noteworthy decrease in the time spent and error numbers in OVX.E2 (*P*<0.01) and OVX. YP (*P*<0.001) groups in Q2 compared to OVX (Figures 4d and e).

### Oxidative system and protein expression

***Oxidative stress markers***

**Figure 5:**
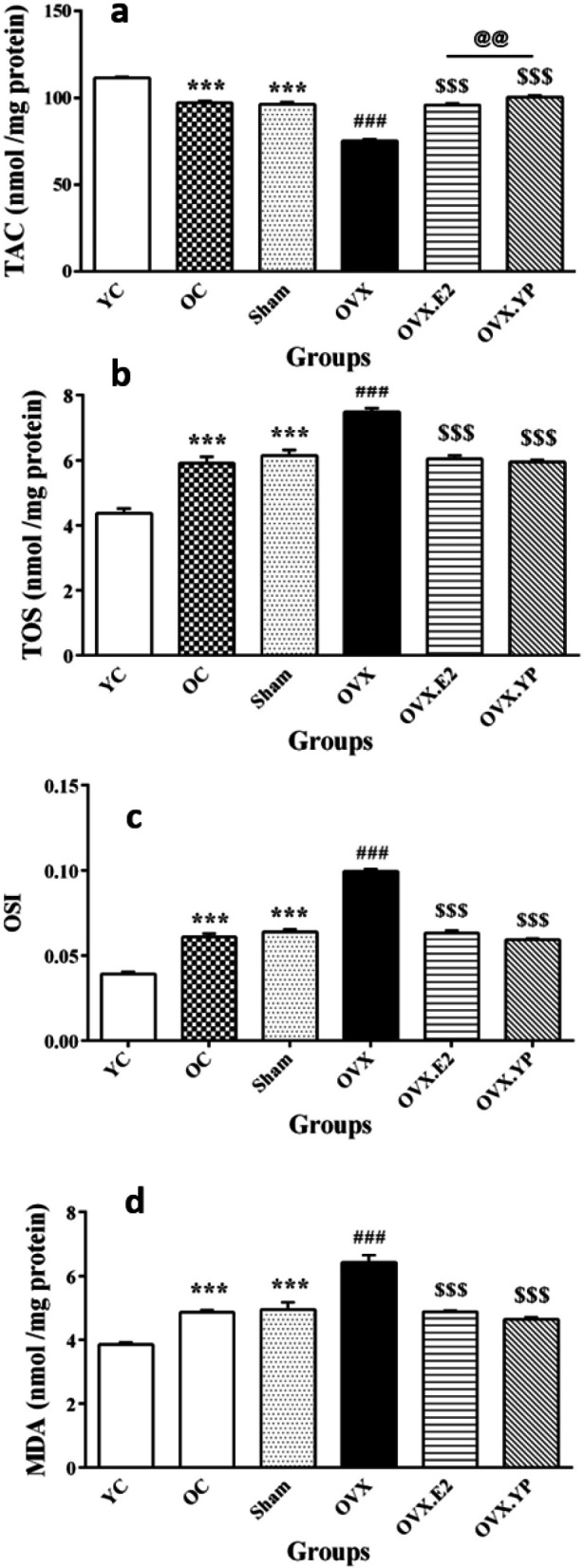
Effect of young plasma on antioxidant markers in hippocampal tissue of ovariectomized old rats

Anti-oxidant system markers were evaluated in the hippocampus tissue, and the results were demonstrated in Figure 5. 

The difference in the hippocampal TAC level reached statistically [F (5, 30) = 187, *P*<0.00010]. 

A significant reduction in TAC was detected in OC and sham groups compared to group YC (*P*<0.001, Figure 5a). TAC was lowered significantly in OVX related to OC and sham (*P*<0.001) while increased meaningfully in OVX.E2 and OVX. YP (*P*<0.001). TAC in OVX. YP was higher than OVX.E2 significantly (*P*<0.01). 

In contrast, based on the obtained results from the analysis of three oxidative factors includes TOS [F (5, 30) = 52.86, *P*<0.0001)], OSI [F (5, 30) = 202.1, *P*<0.0001)] and MDA [F (5, 30) = 36.91, *P*<0.0001)] they were raised significantly in OC and sham groups related to YC (*P*<0.01 and *P*<0.001, Figures 5b-d). Likewise, rats in the OVX group had a notable increase in TOS, OSI, and MDA compared to OC and sham groups (*P*<0.001, Figures 5b-d). Two therapeutic procedures showed that OVX.E2 and OVX. YP groups had a substantial reduction in TOS, OSI, and MDA compared to group OVX (*P*<0.001, Figures 5b-d).

***Western blot findings***

Western blot results revealed the altered measures of p-CREB/CREB [F (5, 30) = 9.723, *P*<0.0001)], BDNF [F (5, 30) = 8.340, *P*<0.0001)] and SIRT1 [F (5, 30) = 21.62, *P*<0.0001)]. 

**Figure 6:**
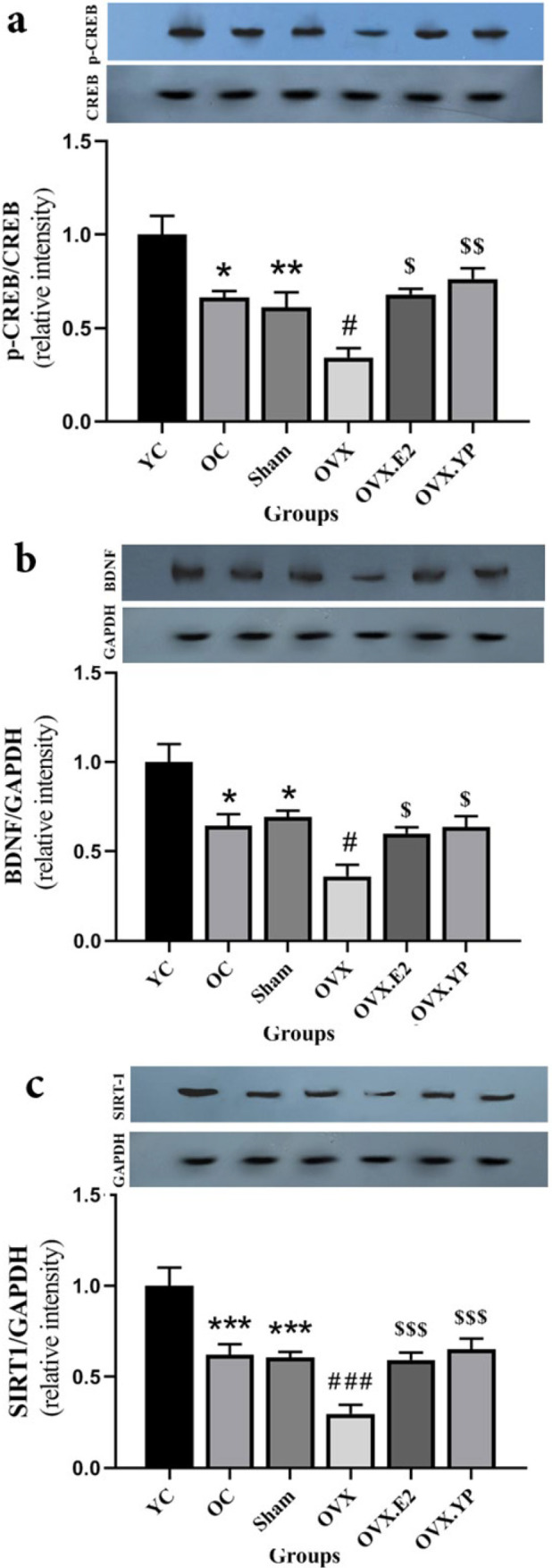
Result of young plasma on the expression stages of p-CREB/CREB, BDNF, and SIRT1 protein expression levels in hippocampal tissue of ovariectomized old rats

Aging markedly decreased hippocampal protein expression of p-CREB/CREB (*P*<0.05 and *P*<0.1 Figure 6a), BDNF (*P*<0.05, Figure 6b), and SIRT1 (*P*<0.001, Figure 6c) in OC and sham groups compared to YC group. As shown in Figure 6, the protein levels of p-CREB/CREB (*P*<0.05), BDNF (*P*<0.05), and SIRT1 (*P*<0.001) in the OVX group were meaningfully decreased compared with OC and sham groups (Figure 6a-c). Following the comparison of OVX.E2 (*P*<0.05) and OVX. YP (*P*<0.01) groups with OVX analyzed data showed that the ratio of p-CREB/CREB increased considerably (Figure 6a), BDNF and SIRT1 were increased significantly (*P*<0.05, Figure 6b, *P*<0.001, Figure 6c). In addition, there was no difference between OVX. YP with OVX.E2 groups for all three above parameters.

***RT-PCR findings***

Based on one-way ANOVA analysis, there was a notable expression alteration between groups for miR-134 [F (5, 30) = 298.9, *P*<0.0001)] and miR-124 [F (5, 30) = 89.70, *P*<0.0001)]. 

**Figure 7:**
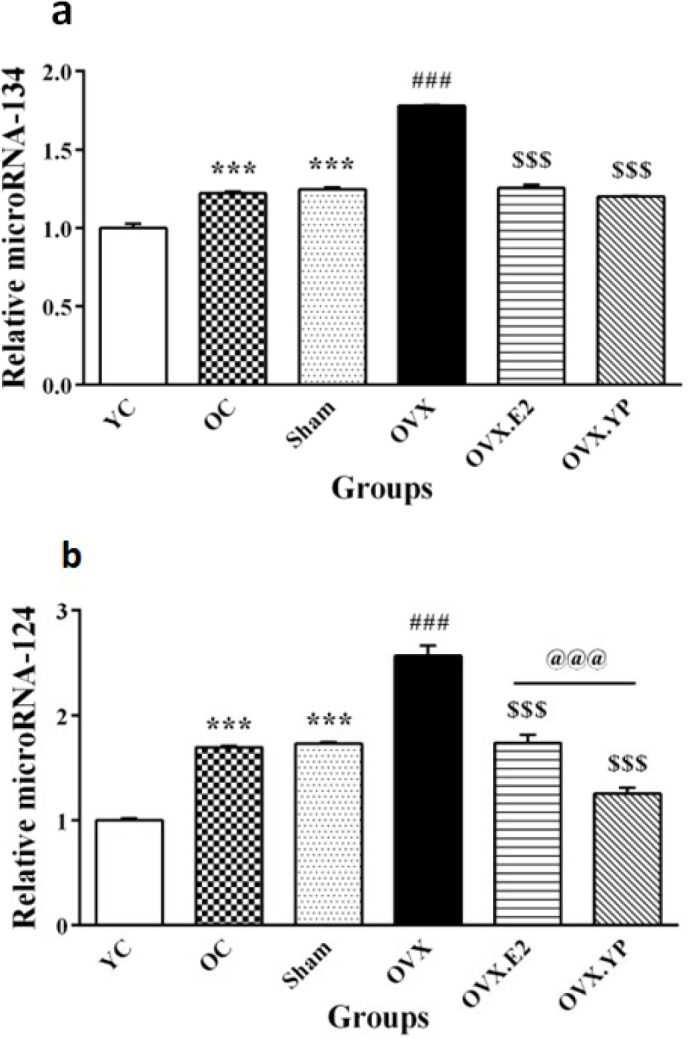
Effect of young plasma on the miR-134 and 124 expressions in the hippocampus of ovariectomized old rats

The expression of miR-134 (*P*<0.001) and miR-124 (*P*<0.001) was reduced in the OC and Sham groups compared to YC (Figures 7a-b). These expressions meaningfully diminished in the OVX group related to OC and sham (*P*<0.001, Figures 7a-b). Additionally, there was a remarkable decrease in the expression levels of miR-134 (*P*<0.001) and miR-124 (*P*<0.001) in the OVX.E2 group compared to OVX (Figures 7a-b). Also, a comparison of OVX. YP group with OVX, a notable decrease was observed in the miR-134 (*P*<0.001) and miR-124 (*P*<0.001) levels. The comparison effect of estrogen and plasma, the appearance level of miR-124 in OVX. YP group was less than associated with OVX.E2 (*P*<0.001), while the expression level of miR-134 did not differ between OVX. YP with OVX.E2.

## Discussion

This study considered the effects of YPT and E-2T on memory and learning in the ovariectomized elderly rats. Our research aimed to examine whether YPT is an efficient and effective alternative to E-2T for reducing the risk of cognitive deficits following ovariectomy or menopause using ovariectomized rats. The important findings of current research are as follows: 1) YPT as much as or more than E-2T restored the decreased cognitive function of OVX rats in OF, EPM, and BMT tests. 2) YPT and E-2T exert the same improving impacts on the hippocampal anti-oxidant system factors toward normal levels. 3) YPT as much as or more than E-2T could normalize the distorted hippocampal proteins BDNF, p-CREB/CREB, and SIRT1 as well as miR-134 and miR-124 gene expression toward the normal levels. 4) YPT and E-2T protected ovariectomy-induced anxiety disorders. 

In this study, ovariectomy surgery was conducted to induce ovarian hormone deficiency in motor and cognitive functions. The findings are in line with previous studies according to which ovariectomy caused anxiety-like and cognitive impairment in rats (3). On the other hand, the beneficial impacts of E2 on the ovariectomized rat were compatible with the findings of recent research about the effect of E2 on neuronal deficits, anxiety, and cognitive impairment in ovariectomized aged rats (19). 

Some evidence suggests that heterochronic parabiosis and young plasma transfusions improve neurogenesis and cognitive function in aged animals (21, 24, 27). The present behavioral tests explicitly demonstrated that the administration of E2, as well as YP, ameliorated not only the locomotor impairment of rats (in the OF test), distress, and anxiety behaviors (in the EPM test) but also improved the spatial learning and memory (in BMT test) in ovariectomized aged rats. These results indicate that YPT exerts protective impacts versus cognitive dysfunction attributable to lack of estrogen or menopause state, a major risk factor for dementia comprising A.D. (27, 40). Moreover, since YPT exerted anti-dementia outcomes that were almost equivalent to or more than the impacts elicited by E2T, this work makes available a rational cause for the use of non-drug treatment for the improvement of anxiety and cognitive disorders. However, whether the circulating blood factors in young plasma may also revitalize the brain performance in aged animal models is yet unknown. Based on conducted recent pilot research on humans, which evaluated the bearableness, safety, and applicability of YPT in aged patients with cognitive disorders and Alzheimer’s disease (A.D.), confirmed that YPT in humans is well endured and does not cause any serious adverse outcomes (41).

The hippocampus is essential in cognitive function, especially anxiety behaviors and spatial memory, and is the brain region most susceptible to oxidative stress damage (42). Oxidative stress and hyper-inflammatory processes produce positive feedback that can be defined as a faulty circle. This raises the development of amyloid genesis and tau hyper-phosphorylation, leading to neuronal deficits, neurodegeneration, and cell death. The mentioned phenomenon has been defined by some researchers to be an important feature related to A.D. (43). To achieve an improved perception of the mechanisms supporting the anti-dementia impacts of YPT and E-2T in ovariectomized old rats, we evaluated the effects of YPT and E-2T on ovariectomy-induced oxidative damage in the hippocampal tissue by measuring TAC, TOS, OSI as an index of oxidative stress, and MDA stages as an index of membrane lipid peroxidation damage in ovariectomized aged rats. Our results showed that a lack of E2 could decrease the TAC level and increase the hippocampus’s TOS, OSI, and MDA levels after YPT and E-2T. It may suggest that the up-regulation of anti-oxidant enzymes and their anti-oxidant defenses is induced by YPT–mediated protection effects (44). Additionally, previous studies reported that mechanisms of estrogen, such as the neuroprotective effect, have focused on the oxidant/anti-oxidant balance, oxidative stress, and inflammation in brain tissue (45). However, the present study did not assess the activity and expression of the anti-oxidant enzyme catalase and the accumulation of reactive oxygen species. The biochemical research is necessary to appreciate better the interplay of reactive oxygen species, enzymatic anti-oxidants, and YPT in cognitive disorders in ovariectomized aged animals. Furthermore, the biological characteristics of blood factors included in the YPT can benefit neurodegenerative disorders for several reasons (46). Following the lack of E2 with regular aging and its outcome on the changing hippocampal integrity and performance, the limited ability of the brain to manage synaptic connections and the developing risk of A.D. happen in menopausal women (47). On the other hand, it has been suggested that SIRT1 ameliorated hippocampal cognition dysfunctions by down-regulation of miR-134 and miR-124, which led to the overexpression of BDNF and p-CREB (45). Thus, many studies have suggested that the appearance of SIRT1, BDNF, and p-CREB proteins in the hippocampal CA1 area can be up-regulated by E-2T (10, 48). According to Scudellari, YPT in older patients effectively relieves cognitive disorders and A.D. symptoms (49). We suppose that the presence of brain growth factors such as BDNF and other hippocampal factors change with the onset of aging and that YPT could affect these types of processes. Therefore, we displayed that the YPT affected the expression of some of these molecules, such as BDNF, p-CREB, and related microRNAs, such as miR-134 and miR-124. They are involved in cognitive impairments, events involving imbalance and anxiety-like behavior, and neuroinflammatory responsive factors, including the SIRT1, had statistically significant higher changes in group OVX. YP, which finally could provide a higher regenerative ability of YPT than E-2T. Further investigations are needed to find the underlying other signaling pathways and mechanisms.

**Table 1:** The sequences of the primers utilized in the RT-PCR

Gene	Target sequence ^a^
U6	5ʹ- TTCGTGAAGCGTTCCATATTTT-3ʹ
rno-miR-124	5′- TAAGGCACGCCGTGAATG -3′
rno-miR-134	5′ - TGTGACTGGTTGACCAGAGGGG -3′

YC: Young Control group, OC: Old Control group. Sham: Sham group, OVX: Ovariectomized group, OVX.E2: The Ovariectomized animals received estrogen and OVX. YP: The Ovariectomized animals received young plasma. Data are accessible as means ± SEM. and analyzed using one-way ANOVA followed by Tukey's *post-hoc* tests (n= 10). * *P*<0.05, ** *P*<0.01, *** *P*<0.001, and **** *P*<0.0001 vs C.Y. group, # *P*<0.05, ## *P*<0.01 and ### *P*<0.001 vs C.O. and Sham groups. $ *P*<0.05, $$ *P*<0.01, and $$$ *P*<0.001 show significant differences between the OVX vs OVX.E2 and OVX. YP groups. @ *P*<0.05, @@ *P*<0.01, and @@@ *P*<0.001 show significant differences between the OVX.E2 and OVX. YP groups.

YC: Young Control group, OC: Old Control group, Sham: Sham group, OVX: Ovariectomized group, OVX.E2: The Ovariectomized animals received estrogen and OVX. YP: The Ovariectomized animals received young plasma. Data are accessible as means ± SEM. and analyzed using one-way ANOVA followed by Tukey's post-hoc tests (n= 10). * *P*<0.05, **P<0.01, *** *P*<0.001, and **** *P*<0.0001 vs C.Y. group, # *P*<0.05, ##* P*<0.01 and ### *P*<0.001 vs C.O. and Sham groups. $* P*<0.05, $$ *P*<0.01, and $$$ *P*<0.001 show significant differences between the OVX vs OVX.E2 and OVX. YP groups. @ *P*<0.05, @@ *P*<0.01, and @@@ *P*<0.001 show substantial differences between the OVX.E2 and OVX. YP groups.

a) Tracking plots and occupancy in the target quadrant (Q2, left-hand lower quadrant) were recorded on the probe day, which indicates the typical tracking plots for the study groups. b) Escape latencies on the three training days showed latency (seconds) in finding the escape. c) Number of errors (incorrect whole investigations) in finding the target box on the three training days. d) Percentage of the time consumed in the target quadrant in the probe trial, e) Number of errors (incorrect hole investigations) on probe day.

YC: Young Control group, OC: Old Control group. Sham: Sham group, OVX: Ovariectomized group, OVX.E2: The Ovariectomized animals received estrogen and OVX. YP: The Ovariectomized animals received young plasma. Data are accessible as means ± SEM. and analyzed using one-way ANOVA followed by Tukey's* post-hoc* tests (n= 10). * * P*<0.05, ** * P*<0.01, *** * P*<0.001, and **** * P*<0.0001 vs C.Y. group, # * P*<0.05, ## * P*<0.01 and ### * P*<0.001 vs C.O. and Sham groups. $ *P*<0.05, $$ *P*<0.01, and $$$ * P*<0.001 show significant differences between the OVX vs OVX.E2 and OVX. YP groups. @* P*<0.05, @@ * P*<0.01, and @@@* P*<0.001 show substantial differences among the OVX.E2 and OVX.YP groups.

YC: Young Control group, OC: Old Control group. Sham: Sham group, OVX: Ovariectomized group, OVX.E2: The Ovariectomized animals received estrogen, OVX.YP. The Ovariectomized animals received young plasma. Data are accessible as means ± SEM. and analyzed using one-way ANOVA followed by Tukey's *post-hoc *tests (n= 10). * *P*<0.05, ** *P*<0.01, *** *P*<0.001, and **** *P*<0.0001 vs C.Y. group, # *P*<0.05, ## *P*<0.01 and ### *P*<0.001 vs C.O. and Sham groups. $*P*<0.05, $$*P*<0.01, and $$$ *P*<0.001 show significant differences between the OVX vs OVX.E2 and OVX. YP groups. @ *P*<0.05, @@ *P*<0.01, and @@@ *P*<0.001 show substantial differences among the OVX.E2 and OVX. YP groups.

a) Ratio of phosphorylation and activation of CREB (p-CREB) expression level to cAMP-responsive element-binding protein (CREB) expression level. b) Ratio of brain-derived neurotrophic factor (BDNF) expression level to GAPDH expression level. c) Sirtuin1 (SIRT1) expression ratio to GAPDH expression level. YC: Young Control group, OC: Old Control group. Sham: Sham group, OVX: Ovariectomized group, OVX.E2: The Ovariectomized animals received estrogen and OVX. YP: The Ovariectomized animals received young plasma. Data are accessible as means ± SEM. and analyzed using one-way ANOVA followed by Tukey's *post-hoc *tests (n= 10). * *P*<0.05, ** *P*<0.01, *** *P*<0.001, and **** *P*<0.0001 vs C.Y. group, # *P*<0.05, ## P<0.01 and ### *P*<0.001 vs C.O. and Sham groups. $ *P*<0.05, $$ *P*<0.01, and $$$ *P*<0.001 show significant differences between the OVX vs OVX.E2 and OVX. YP groups. @ *P*<0.05, @@ *P*<0.01, and @@@ *P*<0.001 show substantial differences amongst the OVX.E2 and OVX. YP groups.

a) Expression levels of miR-134. b) Expression levels of miR-124. YC: Young Control group, OC: Old Control group. Sham: Sham group, OVX: Ovariectomized group, OVX.E2: The Ovariectomized animals received estrogen and OVX. YP: The Ovariectomized animals received young plasma. Data are accessible as means ± SEM. and analyzed using one-way ANOVA followed by Tukey's post-hoc tests (n= 10). * *P*<0.05, ** *P*<0.01, *** *P*<0.001, and **** *P*<0.0001 vs C.Y. group, # *P*<0.05, ##*P*<0.01 and ### *P*<0.001 vs C.O. and Sham groups. $ *P*<0.05, $$ *P*<0.01, and $$$ *P*<0.001 show significant differences between the OVX vs OVX.E2 and OVX. YP groups. @ *P*<0.05, @@ *P*<0.01, and @@@ *P*<0.001 show substantial alterations between the OVX.E2 and OVX. YP groups.

## Conclusion

According to the results, estrogen deficit instigated oxidative stress effects, diminished BDNF, p-CREB, and SIRT1 protein levels, and augmented miR-134a and miR-124 expression in the hippocampus of aged rats. Our results presented that YPT progresses cognition as much as or more than ERT by varying biochemical and molecular signaling factors. This investigation, along with many other studies of different types, concluded that plasma therapy, which is effective but has no side effects compared to hormonal therapy, is a valuable candidate and may be another way to prevent aging, menopause, and other problems such as cognitive impairment. Thus, more studies on other variables and animal models are required before the results of this study can be generalized to humans.

## Data Availability

The data are available for any scientific use with permission.

## Declaration

We have not used any AI tools or technologies to prepare this manuscript.
